# Trauma-Informed Care in Primary Health Settings—Which Is Even More Needed in Times of COVID-19

**DOI:** 10.3390/healthcare8030340

**Published:** 2020-09-14

**Authors:** Teresa Tomaz, Ivone Castro-Vale

**Affiliations:** 1 Medical Psychology Unit, Department of Clinical Neurosciences and Mental Health, Faculty of Medicine, University of Porto, 4200-319 Porto, Portugal; tomaz.teresa@gmail.com; 2 i3S-Institute for Research and Innovation in Health, Rua Alfredo Allen, 208, 4200-135 Porto, Portugal

**Keywords:** traumatic events, clinical communication, patient-centered, post-traumatic stress disorder, somatic comorbidity

## Abstract

Included in the general practitioner’s (GP) core competencies is the ability to adopt a person-centered approach, and the use of the biopsychosocial model in their clinical work. Traumatic events (TEs) are frequently experienced within the population and are known to dysregulate the stress response system and to be associated with psychiatric and physical disorders. GPs may feel reluctant to confront TEs for a variety of reasons, such as a lack of sufficient training in trauma-informed care or a fear of causing harm when discussing a patient’s more complicated issues, among others. This perspective paper aims to review the existing studies that support the practice of trauma-informed healthcare and to summarise best practices. Studies have shown that patients appreciate the questions that clinicians ask them about trauma-related issues and that they understand that this can be important for their healthcare. Furthermore, asking about trauma-related issues in a patient-centered and empathic way can result in better doctor–patient relationships, which improves the levels of satisfaction of both the patient and the doctor with the consultation, as well as improved health-related outcomes. As past traumatic experiences increase the risk of developing post-traumatic stress disorder on exposure to a new TE, the onset of the COVID-19 pandemic has led to trauma-informed care becoming even more important if the strategy is to continue to invest in preventive medicine.

## 1. Introduction

The European definition of General and Family Medicine includes the statement that part of the general practitioner’s (GP) core competencies is the ability to adopt a person-centered approach as well as a holistic perspective, i.e., the ability to use a biopsychosocial model whilst taking into account the cultural and existential facets of patients. Accordingly, GPs should approach their patients’ health problems in such a way that considers all their physical, psychological, social, cultural, and existential dimensions. The longitudinal nature of the care provided in primary health settings places GPs in a privileged position to assess traumatic events (TEs) suffered by their patients, and also their families.

According to the 5th Edition of the Diagnostic and Statistical Manual of Mental Disorders (DSM-5), a TE is experienced when a person is personally exposed to actual or threatened death, serious injury, or sexual violence, or witnesses such events, or when they learn that such an event has happened to someone who is close to them, such as a family member or a close friend [1]. Much of the population has experienced TEs, with a lifetime prevalence ranging from 64% to 90% [2,3]. The onset of the COVID-19 pandemic has become a new source of exposure to several types of TEs (e.g., intensive care unit-related experiences) [4,5]. Although epidemiological data regarding mental health problems related to the COVID-19 outbreak are not yet readily available, this global pandemic presents a serious threat to mental health as it can cause stress-related disorders in patients, their families, and healthcare workers [6,7]. Exposure to a TE triggers the stress system and prepares the person affected to deal with it. The sympathetic nervous system and the hypothalamic–pituitary–adrenal axis enable the person to enact the fight or flight response and to mobilize the energy necessary to deal with the TE. Furthermore, all unnecessary functions are inhibited, such as growth and inflammation by the action of cortisol, which is the main stress system regulator [8]. The dysregulation of this response can lead to many health problems [9]. Furthermore, adverse childhood experiences (ACEs)—which dysregulate the hypothalamic–pituitary–adrenal axis [10]—are prevalent among primary care patients with chronic conditions and have been found to be positively correlated with the number of clinic visits and time to screen, which means that trauma-informed healthcare has become mandatory [11,12,13]. In this perspective paper, the authors aim to review the most relevant studies which support the hypothesis that trauma-informed healthcare is essential for GPs’ clinical practice. Based on this, we propose a model of trauma-informed care best practices.

## 2. Discussion

### 2.1. The Relevance of Traumatic Experiences

Exposure to TEs can result in long-term negative health outcomes, including a greater prevalence of chronic diseases and psychiatric disorders, such as: obesity, post-traumatic stress disorder (PTSD), and depression [14,15]. In particular, PTSD has a high rate of physical and psychiatric comorbidities [14,16,17]. In addition, it has also been shown that exposure to TEs in early life, and even childhood adversity, are both related with an increased risk of developing physical diseases and mental disorders [18,19]. A meta-analysis of 37 studies calculated the risk estimates for 23 outcomes for a total of 253,719 participants, with the objective to understand the relationship between exposure to at least four ACEs and the occurrence of health problems in adulthood, in comparison to those who were not exposed to ACEs [20]. The authors found that those people who had been exposed to at least four ACEs during childhood or adolescence were subject to the increased risk of suffering from ill-health when compared with individuals who had not experienced an ACE. Moderate associations (with odds ratios (ORs) of two to three) were found for smoking, heavy alcohol use, poor self-rated health, cancer, and heart disease; whereas strong associations (with ORs of more than three to six) were recorded for respiratory disease, sexual risk behaviour, mental health problems, and alcohol abuse; and the strongest associations (with ORs of more than seven) were found for drug abuse and interpersonal and self-directed violence. Importantly, the outcomes which are most strongly associated with at least four ACEs represent potential ACE risks for the next generation, such us violence and drug abuse. Additionally, intergenerational effects can also result from ACE-related DNA epigenetic modifications which are inherited, rather than just from parent behaviour-related offspring maltreatment [21,22]. The meta-analysis highlights the need for ACE-informed healthcare services [20].

Delayed onset PTSD and its associated physical comorbidities warrant the need for clinicians to focus on the long-term effects of TEs [23]. Indeed, the continuous assessment of the consequences of TEs is recommended [24]. The diagnosis of PTSD can be a challenge in primary care settings and it was found that only 11% of patients with PTSD diagnosed by means of a structured interview were referenced as such in clinical files [25]. In the past, GPs have underestimated the clinical significance of symptoms when patients with PTSD sought their help [26]. Although TEs are frequently experienced within the population and are known to have negative health consequences, it appears that primary healthcare professionals fail to routinely address this issue with their patients [27]. Some of the reasons pointed out for this lapse include a lack of formal training in communication skills for medical students and continuing education gaps in mental health and trauma training, as well as the fact that clinicians often feel uncomfortable to discuss subjects for which their training has not prepared them (which is analogous to “opening Pandora’s box”) and a fear that the doctor–patient relationship will be harmed and that patients’ privacy will be invaded [28,29]. COVID-19-related traumatic experiences can have two different PTSD-related effects on patients. First, they can be the cause of increased vulnerability to PTSD development in the event of a TE occurring later on; secondly, they can constitute (on their own) the TE which directly causes PTSD, as has been shown to happen in other type of traumatic experiences [30]. Furthermore, COVID-19-related traumatic experiences can cause other stress-related disorders [7].

A study was carried out with the objective to describe the attitudes, capacities, practices, and barriers perceived by a sample of approximately 800 family doctors regarding their screening of adult patients for childhood sexual or physical abuse. The results led to the conclusion that only 29.6% of the doctors surveyed questioned their patients about the occurrence of childhood sexual or physical abuse. Some of the barriers which restrict clinicians from exploring the history of childhood abuse were identified. These barriers include the lack of time to assess or advise people who have gone through these experiences, the lack of skills to do so, and the fear to cause harm to patients or to create an embarrassing environment during the clinical interview [31].

Conversely, some authors found that many patients who have experienced TEs often report negative interactions with healthcare professionals. A qualitative study aiming to understand the perspectives of 23 women with a history of TEs, which included high rates of interpersonal violence, and their interactions with their family doctors [32], found that nearly 60% of patients felt ignored or overlooked by their GP. The same patients also reported experiences of feeling that their concerns were not taken seriously by the healthcare professional and also a lack of empathy. This was particularly evident in the case of women who had an unresolved attachment state of mind, that is to say, women who fall silent in the middle of discussing loss or trauma, or who shift abruptly to another topic. The authors concluded that the experience of trauma in the past can negatively influence patients’ perception of communication with their GP. In addition, GPs can find these patients to be difficult or awkward, which could make it more difficult to establish a positive clinical relationship. Training may help improve the quality of care for trauma patients and attachment-oriented interventions may be useful to help improve trauma-informed care.

### 2.2. Addressing Patients’ Traumatic Experiences

Certain interventions which are designed to ensure that trauma is addressed in primary healthcare settings have been suggested by some authors [32]. These strategies include the training of healthcare professionals in communicating with such patients, in order to enable them to consider the patient’s perspective and use empathy in their clinical interaction. This communication model assumes that clinicians routinely ask their patients if they have already experienced a TE. It is important that it is the clinician who introduces this subject in the consultation, for the simple reason that patients may not feel comfortable starting a conversation about TEs due to feelings of shame, guilt, or a fear that the clinician will not believe them, or even due to PTSD-related avoidance symptoms [33]. Establishing empathetic and trust-based communication enables patients to feel more comfortable in revealing a TE to the healthcare professional.

A cross-sectional study was carried out with the main objective to understand the screening preferences for discussing the trauma histories of primary care patients [34], through the use of a questionnaire, an ACEs survey, and a PTSD screening instrument for primary care settings. The study hypothesized that patients feel more uncomfortable discussing their trauma histories with their clinicians if they manifest one or more ACE or PTSD symptoms than if they have none. The authors of this study also assessed patients’ perceptions of their clinicians’ ability to help them with their traumatic experiences. This study found that 79% of patients felt comfortable about being asked about ACEs and PTSD symptoms, or about being screened for these events (86%), regardless of whether they had experienced any type of TE. In addition, 73% of patients surveyed believed that their clinicians could help them with the ACEs they had been exposed to. The authors concluded that most patients feel comfortable about being questioned directly by their clinicians or through the use of a screening survey enquiring about past traumatic experiences, regardless of the number of experiences. Most patients were comfortable about the results of their screening survey being recorded in their medical records. This study suggests that patients not only want to be asked about TEs, but also expect clinicians to routinely question them, especially if this information and an understanding can help improve the treatment of their health problems, which highlights the importance of considering patient preferences when implementing trauma-informed care in a patient-centered way.

Regarding the increasingly recognized importance of asking patients about TEs, several trauma-related communication models [12,13,35] have been developed in primary healthcare settings, such as the Trauma-Informed Medical Care model [12,13]. These models are based on patient-centered approaches, with the aim of developing a relationship between the healthcare professional and the patient that is based on trust and on the construction of a comfortable and private environment in which to discuss trauma-related issues. These models also focus on promoting patient autonomy and on multidisciplinarity, that is to say, the referral of such patients to trauma-specialized mental health professionals. However, this model of approach to patients’ traumatic experiences in primary health care does not intend to cure trauma, nor does it imply that the GP is a specialist in trauma. Instead, it promotes a trusting environment for patients to discuss trauma-related issues with their physicians. Although the existing literature on the effectiveness of these communication models is still scarce, Green et al. [13] show that subjecting GPs to a Trauma-Informed Primary Care communication course increased the simulated patient-centeredness perception from pre- to post-training, which supports the hypothesis that GPs’ relationship-based trauma-informed care could help promote better patient health-related outcomes. Subsequently, a pilot study applied this six-hour CME training course for GPs to real patients in order to examine whether they were awarded a higher rating following the training of their GPs. The training did indeed increase the positive aspects of the GP–patient partnership from both observer ratings and patient perspectives, particularly in the areas of comfort and partnership, although specific trauma-related outcomes were not included [12].

In this research, we have evidenced the need for trauma-informed care communication courses for GPs. Based on the studies reviewed, the authors suggest the following model for GP trauma-informed care best practice:

(1) *Screen and recognise traumatic experiences*: to ask patients about their exposure to trauma in a private and safe setting and by showing availability, with the use of active listening to discuss the subject, whilst also recognising patients’ experiences; using an empathic posture and a non-judgmental attitude which respects patients’ preferences for disclosure and acknowledge that disclosure is difficult.

(2) *Respond*: by using a patient-centered communication model whereby patients disclose traumatic experiences and by providing a trusting doctor–patient relationship. Employ psychoeducation regarding the effects of trauma on health and health-related behaviours in order to help provide comfort and empowerment to patients. Patients should also be allowed to collaborate in decisions in order to maximise their autonomy and show them respect.

(3) *Minimise suffering and promote resilience*: by providing emotional safety and by being able to adequately respond to strong emotions. Avoid the use of stimuli which increase anxiety or re-experiencing, as well as any other re-traumatisation. It is also important to encourage resilience by informing the patient about treatment options whenever needed, and, if applicable, focus on the positive aspects of the patient’s life.

(4) *Reference*: by referring patients to a specialised mental health services according to their needs (such as symptoms of anxiety, depression, or PTSD) and preferences; this multidisciplinary collaboration may also include other supports, such as the service of social workers.

## 3. Conclusions

TE or ACE exposure history is a frequent reality which is still under-recognized in primary health care. It affects many patients, as these traumas lead to significant negative impacts on health. Patients who have experienced past TEs frequently have disclosure difficulties and often feel misunderstood by their physicians. Accordingly, the starting point for empathic communication with these patients is to identify whether they have experienced past TEs. The studies carried out in this area to date indicate that patients want to be asked about TEs, and that this can improve the patient–physician therapeutic alliance and consequently lead to better healthcare outcomes. Past studies have shown that the establishment of a good GP–patient relationship should consider discovering patients’ preferences for past trauma disclosure.

Furthermore, the importance of addressing past TEs is becoming increasingly recognized, as such TEs tend to increase the probability of the onset of PTSD on exposure to another TE [36]. Unfortunately, the COVID-19 pandemic is exposing many individuals to an enormous traumatic burden which is characterised by different types of TEs, ranging from the loss of a family member through to intensive care unit-related experiences [37]. TE exposure is the cause of many psychiatric and physical health problems and PTSD on its own is associated with many somatic comorbidities. To prevent further suffering, it is even more imperative nowadays that clinicians approach their patients from a patient-centered integrated perspective which includes trauma-informed care. Indeed, the COVID pandemic represents a new challenge for the GP–patient relationship, which cannot be disregarded, considering that the long-term health-related costs could well be too high. Tele-health approaches for the treatment of PTSD have been proposed [38].

Further studies are needed to better understand the impact of trauma-informed patient-centered communication in primary health care settings in several domains, such as patient perceptions and satisfaction, as well as health-related outcomes.
